# Supplementary material for: MetaRibo-Seq measures translation in microbiomes
Source: Nat Commun. 2020 Jun 29;11:3268. doi: 10.1038/s41467-020-17081-z (PMC7324362; doi:10.1038/s41467-020-17081-z)
Supplement: Supplementary file 10 — Supplementary Data 7 [file 41467_2020_17081_MOESM10_ESM.zip › File2/Confidence_High_Taxonomy/134295_out.krona.html]

Javascript must be enabled to view this page.

members
magnitude
magnitudeUnassigned
count
unassigned
taxon
rank

134295\_out

9

2
superkingdom
9

9
phylum
1239

186801
class
9

186802
9
order

family
2
31979

2
genus
1485

2

SRS013940\_contig\_number\_contig-100\_10083.62851SRS1054691\_contig\_number\_10789
species
59620

541000
family
7

1263
7
genus

species
7

SRS015890\_contig\_number\_9207SRS048060\_contig\_number\_2091SRS050752\_contig\_number\_20711SRS051610\_contig\_number\_contig-100\_377.38470SRS052697\_contig\_number\_37989SRS074964\_contig\_number\_contig-100\_1454.50197SRS142542\_contig\_number\_24871
46228
